# Development of a Novel 11-Gene Signature Related to Immune Subtypes for Fibromyalgia

**DOI:** 10.2174/0118715303365068250303042017

**Published:** 2025-03-10

**Authors:** Wei Zhao, Pengcheng Wang

**Affiliations:** 1 Department of Anesthesiology, The First Affiliated Hospital of Harbin Medical University, Harbin, 150001, China;; 2 Department of Colorectal Surgery, Shanxi Province Cancer Hospital/Shanxi Hospital Affiliated to Cancer Hospital, Chinese Academy of Medical Sciences/Cancer Hospital Affiliated to Shanxi Medical University, Taiyuan, 030000, China

**Keywords:** Fibromyalgia, immune-related genes, molecular subtypes, hub genes, diagnosis, transcription factor, immune cell infiltration

## Abstract

**Aim:**

The purpose of this study was to identify molecular subtypes and hub genes in fibromyalgia (FM) based on immune-related genes (IRGs).

**Background:**

FM is a chronic disease featuring widespread pain, and the immune system may be involved in the FM progression.

**Objective:**

The objectives of this study are as follows: 1) To identify the molecular subtypes of FM based on IRGs. 2) To screen and validate the hub genes in FM. 3) To predict the transcription factor (TF) targeting hub genes and 4) To evaluate the correlation between immune cell infiltration, hallmark pathways, and hub genes.

**Methods:**

Two FM datasets were acquired from the Gene Expression Omnibus (GEO) database. IRGs were collected from the ImmPort database. Molecular subtypes of FM were identified using the “ConsensusClusterPlus” package. IRGs score and differentially expressed genes (DEGs) between different FM subtypes and control samples were obtained using “GSVA” and “limma” packages. Key module genes related to FM subtypes were identified using the “WGCNA” package. Hub genes were screened and verified using “glmnet” and “pROC” packages. TF-hub gene regulatory network was constructed by Cytoscape software. The correlation between immune cells, hallmark pathways, and hub genes was analyzed by the Spearman method. Finally, the DSigDB database was used to obtain associations between characterized genes and drugs, and the expression of key genes was verified using qRT-PCR.

**Results:**

FM samples were classified into two subtypes, and the IRGs score of the C2 subtype was lower than that of the C1 subtype. Then, 184 module genes were obtained and mainly enriched in immune-related pathways. Next, 11 hub genes (*TSPAN16*, *RILPL2*, *RASSF5*, *PGAP2*, *PADI2*, *NACC1*, *LRRC25*, *ITGAD*, *HIPK1*, *ATP6V0D1*, *AP1M2*) were screened with good diagnostic performance. Besides, 45 TFs targeting hub genes were predicted. Most hub genes were negatively associated with CD4/CD8 T cells while positively correlated with macrophages, mast cell, monocyte, and neutrophil, as well as inflammatory response, angiogenesis pathways, *etc*. Molecular docking suggests that chloroquine and L-citrulline may be potent agents binding to *NACC1* and *PADI2*. *RILPL2* and *ITGAD* were significantly differentially expressed in FM-modeled mice.

**Conclusion:**

This study identified two subtypes and 11 hub genes of FM based on IRGs, providing a reference for the clinical diagnosis of FM.

## 1 INTRODUCTION

Fibromyalgia (FM) is categorized as a primary and widespread pain illness by the International Classification of Diseases 11^th^ revision [1]. After osteoarthritis, FM is the second most prevalent rheumatism, with an incidence rate of 1% to 5% in the ordinary population [2]. FM is usually accompanied by complicated multi-symptoms, including fatigue, cognitive impairments, and neurological and mental disorders, in light of which FM patients might suffer from an increase in mortality rate [3]. The pathogenesis of FM is not completely known, but genetic, inflammatory, immune, endocrine, and social psychology are considered crucial factors affecting FM progression [4]. The therapeutic strategies for FM involve multiple disciplines, such as lifestyle adjustment, nutritional supplements, drug treatment, and cognitive-behavioral therapy [5]. At present, the diagnostic criteria of FM depend on integrated scores of widespread pain index (WPI) and symptom severity scale (SSS) [6]. However, the reliable and effective diagnostic biomarkers are very limited, as well as FM is a non-isolated syndrome, resulting in a raised possibility of misdiagnosis and delayed care, which is a core obstacle for the early diagnosis and treatment of FM [7]. Thus, developing a novel and accurate biomarker is of great importance for improving the prognosis and life quality of FM patients and may supply some references for exploring the pathogenesis of this syndrome [8].

A growing number of studies suggest that the immune system, comprising interleukins (IL), chemokines, and immune-regulatory cytokines, plays a crucial role in the development and progression of FM [9, 10]. For example, Gerdle *et al*. manifested that the plasma proteins from the constituents of the immune system could distinguish FM patients from controls by integrating targeted and non-targeted proteomics [11]. Andrés-Rodríguez *et al*. elaborated on the immune variations in FM patients compared with controls and found that IL-6, IL-4, and IL-17A were markedly increased in FM, together with the upregulated M1 macrophage [12]. Mast cells, monocytes, and neutrophils, were also guessed to be involved in the formation of FM inflammatory matrix [13], yet its specific mechanism remained unknown. Immunophenotypic analysis of patient blood samples uncovered that the Mu opioid receptor on B lymphocytes could serve as a particular biomarker for FM [14]. Additionally, the genes that encode the pro-inflammatory molecules generated by innate immune system cells have been recognized as a signature for FM patients with depression [15]. These findings support that regulating the immune system may be a promising treatment direction for FM [16]. Therefore, exploring immune-related genes (IRGs) in FM is of great importance for understanding its pathogenesis and developing potential therapeutic targets.

In this study, we identified the molecular subtypes and hub genes in FM based on IRGs, providing some reference for the clinical diagnosis of FM. Two FM datasets were collected from the Gene Expression Omnibus (GEO) database. The molecular subtypes of FM were classified based on IRGs by consensus clustering analysis. Then, the hub genes were screened by weighted gene co-expression network analysis (WGCNA) and least absolute shrinkage and selection operator (LASSO) regression analysis. Meanwhile, the diagnostic values of hub genes were verified in the external dataset. The transcription factor (TF) targeting hub genes was predicted, and the correlation between immune cell infiltration, hallmark pathways, and hub genes was analyzed by the Spearman method.

## 2 MATERIALS AND METHODS

### 2.1 Data Acquisition

The gene expression data of GSE67311 and GSE221921 were downloaded from the GEO database (https://www.ncbi.nlm.nih.gov/geo/) [17]. The GSE67311 dataset was utilized as the training set, comprising 67 FM samples and 75 control samples. The GSE221921 dataset was used as the validation set, containing 96 FM samples and 93 control samples.

### 2.2 Identification of Molecular Subtypes of FM

Firstly, the expression data of IRGs were collected from the ImmPort database (https://www.immport.org/shared/) [18], including 2483 IRGs. Then, the molecular subtypes of FM samples in the GSE67311 dataset were identified based on IRGs by consensus clustering analysis using the “ConsensusClusterPlus” R package [19]. The “km” algorithm and “1-Spearman correlation” were applied as the metrics for distance. The sampling was conducted on 500 bootstraps, and each bootstrap involved 80% of patients. The threshold of clustering (k) was set from 2 to 10. Afterward, principal component analysis (PCA) was performed to assess the reasonability of the subtype classification [20].

### 2.3 Analysis of Differentially Expressed Genes (DEGS)

The IRGs score between different FM subtypes and control samples in the GSE67311 dataset was compared by single sample gene set enrichment analysis (ssGSEA) using the “GSVA” R package [21]. Then, the DEGs between different FM subtypes and control samples were screened using the “limma” R package (*p*<0.05) [22].

### 2.4 WGCNA

The key module genes related to FM subtypes were identified in the GSE67311 dataset using the “WGCNA” R package [23]. In brief, the optimal soft threshold (β) was determined using the pickSoftThreshold function to ensure that the network was a scale-free network. Then, average-linkage hierarchical clustering was performed, and the modules with similar expressions were merged (height=0.25, deepSplit=2, and minModuleSize=100). The ssGSEA score and FM subtypes were utilized as traits, and the module-trait relationships were analyzed by the Spearman method and displayed by a heatmap. Next, the key module with the highest correlation coefficient was obtained, and the correlation between gene significance (GS) and module membership (MM) in the key module was analyzed. Subsequently, the genes in the key module with GS>0.3 and MM>0.3 were selected to intersect with the DEGs between FM subtypes and control samples, ultimately obtaining the module genes related to FM subtypes.

### 2.5 Gene Set Enrichment Analysis (GSEA)

In order to understand the biological functions associated with FM subtypes and module genes, the Kyoto Encyclopedia of genes and genomes (KEGG) and gene ontology (GO) in the biological process (BP) enrichment analysis were conducted by GSEA with the “clusterProfiler” R package (*p*<0.05) [24]. Meanwhile, the top 10 KEGG pathways of normalized enrichment scores (NES) were screened from the different subtypes [25].

### 2.6 Screening and Validation of Hub Genes

We performed LASSO regression analysis (5-fold cross-validation) on 184 candidate genes using the “cv.glmnet” function from the glmnet package, setting the parameter to “lambda.min = 0.05” and obtaining the characterized genes [26, 27]. Next, based on the characterized genes screened, we used the (glm) function in R to fit a logistic regression model, which helps to distinguish FM from normal control samples. Then, the receiver operating characteristic (ROC) curves were established in the GSE67311 and GSE221921 datasets by plotting the sensitivity against specificity using Logistic regression with the “pROC” R package [28]. The diagnostic values of hub genes in distinguishing FM from controls were evaluated by the area under the ROC curve (AUC). Besides, the expression levels of hub genes were compared between FM and control samples.

### 2.7 Construction of Tf Regulatory Network

The possible TFs targeting the hub genes were predicted using the NetworkAnalyst online tool (https://www.networkanalyst.ca/) through the JASPAR database (https://jaspar.elixir.no/) [29]. Then, the TF-hub gene regulatory network was visualized by the Cytoscape software [30].

### 2.8 Correlation Between Immune Cell Infiltration, Pathways, and Hub Genes

The enrichment scores of 28 types of immune cells in each sample were calculated using ssGSEA, and the gene sets of the 28 types of immune cells were obtained from previous literature [31]. Meanwhile, the hallmark pathways of h.all.v2023.2.Hs.symbols.gmt were acquired from the GSEA website (http://software.broadinstitute.org/gsea/ index.jsp), and the enrichment scores of each pathway were computed by ssGSEA using the “GSVA” R package [32]. Then, the correlation between immune cell infiltration, pathways, and hub genes was analyzed by the Spearman method.

### 2.9 Potential Therapeutic Drugs Prediction and Molecular Docking

We first used the Enrichr package for drug prediction of the screened hub genes with the DSigDB database (https://dsigdb.tanlab.org/DSigDBv1.0/). Based on the results of drug prediction, we selected specific drug molecules and downloaded (https://pubchem.ncbi.nlm.nih.gov/) the 3D structures of the drug molecules from the PubChem website and used Chem3D software to minimize the drug structure energy. Before starting molecular docking, all molecules were dehydrogenated, hydrogenated, and removed from small molecules using pymol and AutoDocktools software; subsequently, receptor protein-drug molecular docking was performed using AutoDock Vina. Finally, the crystal structures of specific receptor proteins were obtained through the PDB database.

### 2.10 Animals and Fm Model Design

All experimental protocols adhered to the Guide for the Care and Use of Laboratory Animals of the US National Research Council and the ARRIVE guidelines as appropriate. Our current study has obtained the approval from the Ethics Committee for Animal Experimentation of Zhejiang MEBOLO Biotechnology CO., Ltd (approval no. AL_JMBL2023_006).

A total of twelve female C57BL/6 mice (6-8 weeks), each weighing approximately 20 ± 3 g, served as the subjects for this experimental study and were obtained from Shanghai SLAC Laboratory Animal Co., Ltd. These mice were kept in plastic cages, with six individuals per cage. They were maintained under controlled environmental conditions, including a light cycle of 12 hours of light followed by 12 hours of darkness and a temperature regulated at 25 ± 2 ◦C. During the study, the mice had unrestricted access to standard food pellets and tap water. Prior to the experimentation, the animals underwent an acclimatization period lasting one week.

The mice were randomly separated into two distinct groups, namely, control and FM. The mice of the control group received intravenous physiological saline over a period of two weeks. In contrast, the mice of the FM model group were subcutaneously administered with reserpine (1 mg/kg) for three days, followed by the administration of physiological saline for the subsequent two weeks.

### 2.11 Sample Collection and Gene Expression Testing

Total RNA from mouse gastrocnemius muscle tissue was extracted with TRIzol (Invitrogen, Carlsbad, CA, USA), and cDNA was synthesized using the SuperScript™ III Reverse Transcriptase Kit (Invitrogen, USA). Quantitative reverse transcription PCR (qRT-PCR) was conducted using an ABI Prism 7500 method with SYBR Green qPCR Master Mix (Takara, Shanghai, China). The conditions for qPCR were set at 94°C for 30 seconds, followed by 40 cycles of 94°C for 5 seconds and 60°C for 30 seconds. The relative expression levels of *RILPL2* and *ITGAD* were evaluated through the 2^−ΔΔCt^ approach, utilizing β-actin as a reference standard. All experiments were carried out in triplicate. Primer sequences were as follows:

*RILPL2*, Forward: 5′- CAAAATGGTGGTTGACCTGACA -3′;

Reverse: 5′- GGAGCTGCGACTTGAGTTTGT -3′;

*ITGAD*, Forward: 5′- GCATCATCCGCTACGCTATC -3′;

Reverse: 5′- GGCTGCAAAGTTGTCCACCT -3′;

β-actin, Forward: 5′-GGACATCCGCAAAGACCTGTA -3′;

Reverse: 5′- GCTCAGGAGGAGCAATGATCT -3′.

### 2.12 Statistical Analysis

All statistical analyses were conducted in the R language (version 4.0.3). One-way analysis of variance (ANOVA) test and Wilcoxon rank test were used to determine the statistical difference. Student's t-test was used to compare the differential expression analysis of genes between the control and FM groups. The significance was analyzed on GraphPad Prism (version 8.0). All data were presented as mean± standard deviation (SD). *p*<0.05 was viewed to be statistically significant.

## 3 RESULTS

### 3.1 Two Molecular Subtypes of FM were Identified Based on IRGs

**Fig. (1):**
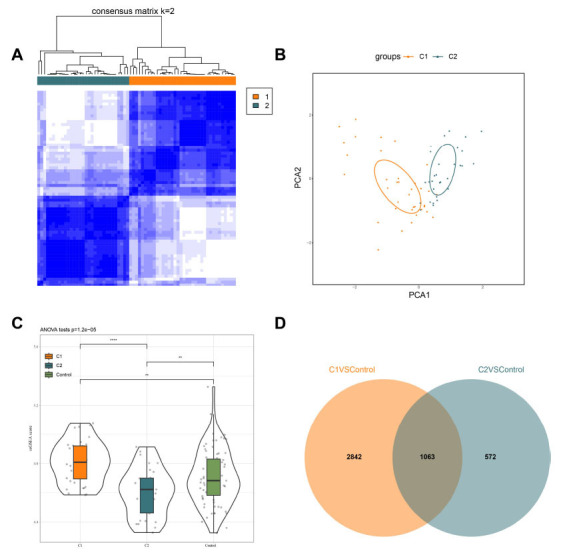
Identification of molecular subtypes in fibromyalgia (FM) based on immune-related genes (IRGs). (**A**) Consensus clustering heatmap of FM samples in GSE67311 dataset; (**B**) Principal component analysis (PCA) of FM subtypes; (**C**) IRGs score between different FM subtypes and control samples calculated by ssGSEA; **** means *p* <0.0001; ** means *p* < 0.01; (**D**) Venn diagram of differentially expressed genes (DEGs) between different FM subtypes and control samples.

Based on the expression data of IRGs, the FM samples in the GSE67311 dataset were separated into two molecular subtypes with clustering stability k=2 (Fig. **1A**). PCA showed that there were obvious boundaries between the C1 and C2 subtypes (Fig. **1B**), indicating that the clustering classification was reasonable. Then, the IRG scores between different FM subtypes and control samples were compared by ssGSEA. It was found that the IRGs score of the C2 subtype was notably lower, while the C1 subtype was markedly higher than control (Fig. **1C**). Furthermore, a total of 3905 DEGs between the C1 subtype and control, as well as 1635 DEGs between the C2 subtype and control, were obtained by intersecting which 1063 DEGs related to FM subtypes were screened for subsequent analysis (Fig. **1D**).

### 3.2 C1 and C2 Subtypes of FM were Enriched in Different KEGG Pathways

**Fig. (2):**
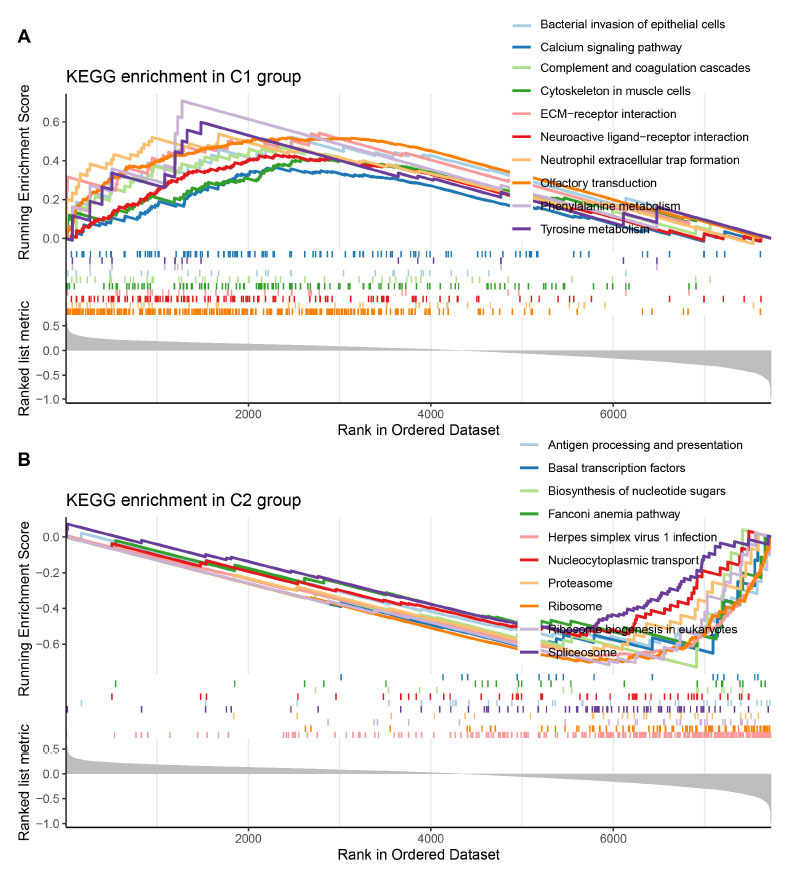
Gene set enrichment analysis (GSEA) of different FM subtypes. (**A**) KEGG enrichment pathways in C1 subtype; (**B**) KEGG enrichment pathways in C2 subtype.

To further understand the biological pathways and molecular mechanisms relevant to FM subtypes, KEGG enrichment analysis was performed by GSEA. C1 subtype was mainly enriched in the pathways of Complement and coagulation cascades, Cytoskeleton in muscle cells, ECM-receptor interaction, Neutrophil extracellular trap formation, Phenylalanine metabolism, Tyrosine metabolism, *etc*. (Fig. **2A**). Whereas the C2 subtype was chiefly enriched in the pathways of Antigen processing and presentation, Basal transcription factors, Biosynthesis of nucleotide sugars, Nucleocytoplasmic transport, Proteasome, Ribosome, Spliceosome, and so on (Fig. **2B**). These results indicated that FM patients in the C1 subtype might experience a high immune activity, yet the C2 subtype may suffer from immune dysfunction.

### 3.3 WGCNA Screened 184 Module Genes Associated with FM Subtypes

**Fig. (3):**
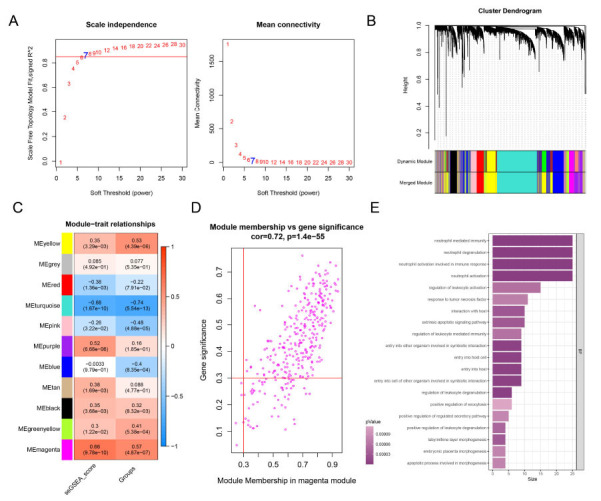
Screening of module genes associated with FM subtypes by WGCNA in GSE67311 dataset. (**A**) Determination of soft threshold (β) to construct topology networks; (**B**) Cluster dendrogram of all genes; (**C**) Heatmap of module-trait relationships; (**D**) Scatter plot of gene significance (GS) *versus* module membership (MM) in magenta module; (**E**) GO enrichment terms of module genes in biological process (BP).

First, the critical module related to FM subtypes was identified by WGCNA. The soft threshold (β) was selected as 7 to construct topology networks (Fig. **3A**). Then, a hierarchical cluster dendrogram was built, obtaining 11 gene co-expression modules (Fig. **3B**). Module-trait relationships heatmap suggested that the magenta module exhibited strong correlation with IRGs score (cor=0.66) and FM subtypes (cor=0.57) (Fig. **3C**). Thus, the genes in the magenta module were used to analyze the correlation between GS and MM (cor=0.72) (Fig. **3D**). And 240 genes in the magenta module with GS>0.3 and MM>0.3 were selected to intersect with the 1063 DEGs between FM subtypes and control samples, ultimately screening 184 module genes associated with FM subtypes. Furthermore, GO enrichment analysis showed that these module genes were significantly enriched in immune-related BP terms, such as neutrophil-mediated immunity, neutrophil activation involved in immune response, regulation of leukocyte-mediated immunity, and regulation of leukocyte degranulation (Fig. **3E**).

### 3.4 11 Hub Genes were Identified and Validated

**Fig. (4):**
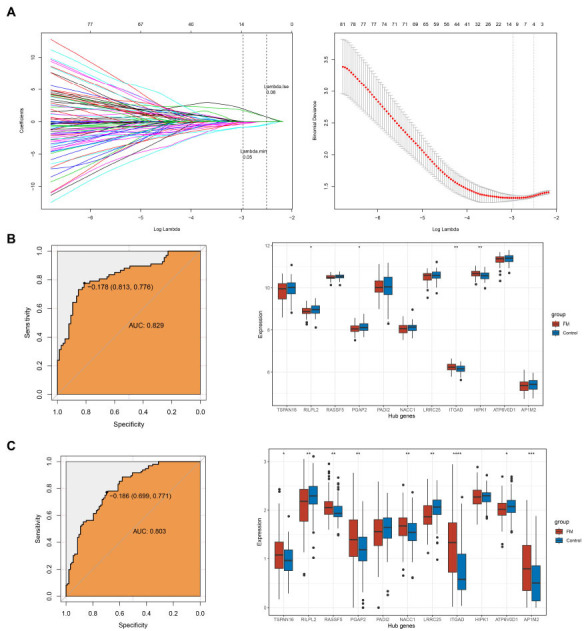
Identification and verification of hub genes in FM. (**A**) The optima lambda value in LASSO regression analysis; (**B**) ROC curves and expression levels of hub genes in the GSE67311 dataset; (**C**) ROC curves and expression levels of hub genes in the GSE221921 dataset; **** means *p* < 0.0001; *** means *p* < 0.001; ** means *p* < 0.01; * means *p* < 0.05.

LASSO regression analysis was performed on the 184 module genes, and the model with lambda.min=0.05 was selected to obtain 11 hub genes (Fig. **4A**), including *TSPAN16*, *RILPL2*, *RASSF5*, *PGAP2*, *PADI2*, *NACC1*, *LRRC25*, *ITGAD*, *HIPK1*, *ATP6V0D1*, and *AP1M2*. Further, the predictive performance of 11 hub genes in distinguishing FM from controls was evaluated and verified by ROC curves. The AUC value of 11 hub genes in the GSE67311 dataset was 0.829 (Fig. **4B**), and in the GSE221921 dataset was 0.803 (Fig. **4C**), which revealed that these hub genes exhibited high sensitivity and specificity for FM diagnosis. Moreover, the expression levels of 11 hub genes were compared between FM and control samples. In the GSE67311 and GSE221921 datasets, *ITGAD* was all highly expressed while *RILPL2* was all lowly expressed in FM samples (Fig. **4B** and **C**), manifesting that *ITGAD* and *RILPL2* may exert a crucial role in the progression of FM.

### 3.5 TF-hub Gene Regulatory Network was Established

**Fig. (5):**
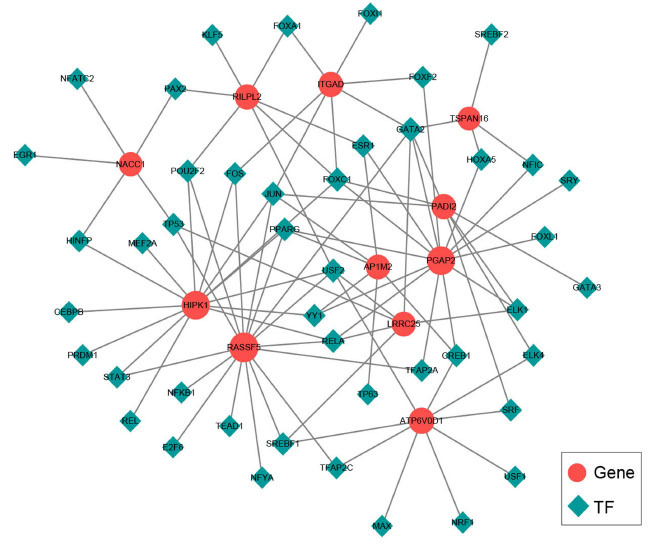
Transcription factor (TF) regulatory network diagram of hub genes. The red dots represent hub genes and blue diamonds represent TFs. Transcription factor (TF) regulatory network diagram of hub genes. The red dots represent hub genes and blue diamonds represent TFs.

A total of 45 TFs targeting the 11 hub genes were predicted to construct a TF-hub gene regulatory network (Fig. **5**). Notably, *TSPAN16* targeted 4 TFs, including GATA2; *RILPL2* targeted 7 TFs, including FOXC1; *RASSF5* targeted 16 TFs, including PPARG; *PGAP2* targeted 14 TFs, including RELA; *PADI2* targeted 9 TFs, including JUN; *NACC1* targeted 5 TFs, including TP53; *LRRC25* targeted 6 TFs, including USF2; *ITGAD* targeted 7 TFs, including FOXA1; *HIPK1* targeted 14 TFs, including POU2F2; *ATP6V0D1* targeted 9 TFs, including USF2; and *AP1M2* targeted 6 TFs, including ESR1.

### 3.6 The Correlation between Immune Cell Infiltration, Pathways, and Hub Genes was Analyzed

**Fig. (6):**
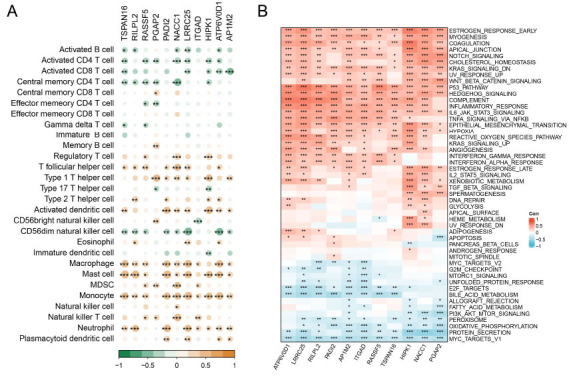
Transcription factor (TF) regulatory network diagram of hub genes. The red dots represent hub genes and blue diamonds represent TFs. (**A**) Correlation heatmap between 28 immune cells and 11 hub genes; (**B**) Correlation heatmap between hallmark pathways and 11 hub genes; *** means *p* < 0.001; ** means *p* < 0.01; * means *p* < 0.05.

The correlation heatmap between 28 types of immune cells and 11 hub genes showed that most hub genes were negatively associated with Activated B cell, Activated CD4 T cell, Activated CD8 T cell, central memory CD4 T cell, and CD56dim natural killer cell, while positively correlated with T follicular helper cell, Type 1 T helper cell, Activated dendritic cell, macrophage, mast cell, monocyte, and neutrophil (Fig. **6A**). In addition, the correlation heatmap between hallmark pathways and 11 hub genes suggested that most hub genes were positively related to the P53 pathway, IL6 JAK stat3 signaling, epithelial-mesenchymal transition (EMT), angiogenesis, and transforming growth factor (TGF) beta signaling, while negatively relevant to the MYC targets V1, protein secretion, oxidative phosphorylation, peroxisome, bile acid metabolism, and E2F targets (Fig. **6B**).

### 3.7 Drug Prediction and Molecular Docking

**Fig. (7):**
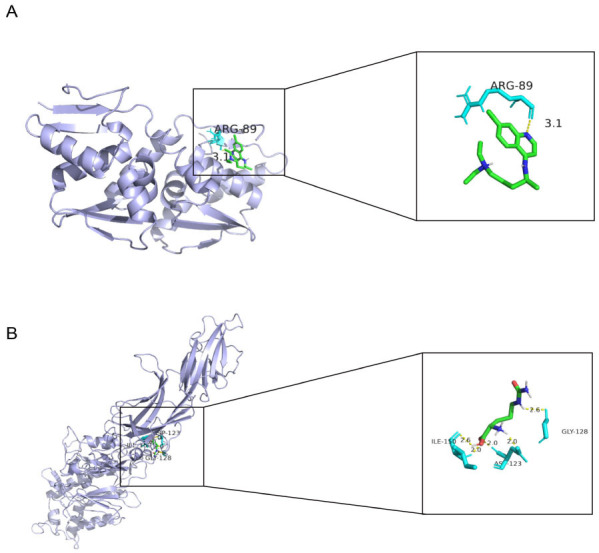
Potential therapeutic agents and conducting molecular docking. (**A**) Diagrammatic representation of the molecular docking configuration of NACC1 with chloroquine; (**B**) Diagrammatic representation of the molecular docking conformation of PADI2 with L-citrulline. The bright blue molecule on the left side of the figure is the receptor protein, the green molecule is the drug small molecule, and the lime green is the amino acid. The hydrogen bond between the receptor protein and the small drug molecule is shown by the yellow dotted line, and the number above it is the length of the hydrogen bond in angstroms (Å).

Based on the 11 hub genes screened, we obtained two target drugs, chloroquine, and L-citrulline, from the DSigDB database and the Enrichr package (Table **1**). Subsequently, we obtained the crystal structures of two receptor proteins, *NACC1* and *PADI2*, from the PDB database and performed receptor protein-drug molecule docking (Figs. **7A** and **B**).

### 3.8 qRT-PCR-based Validation on the Expression of Key Genes in FM

**Fig. (8):**
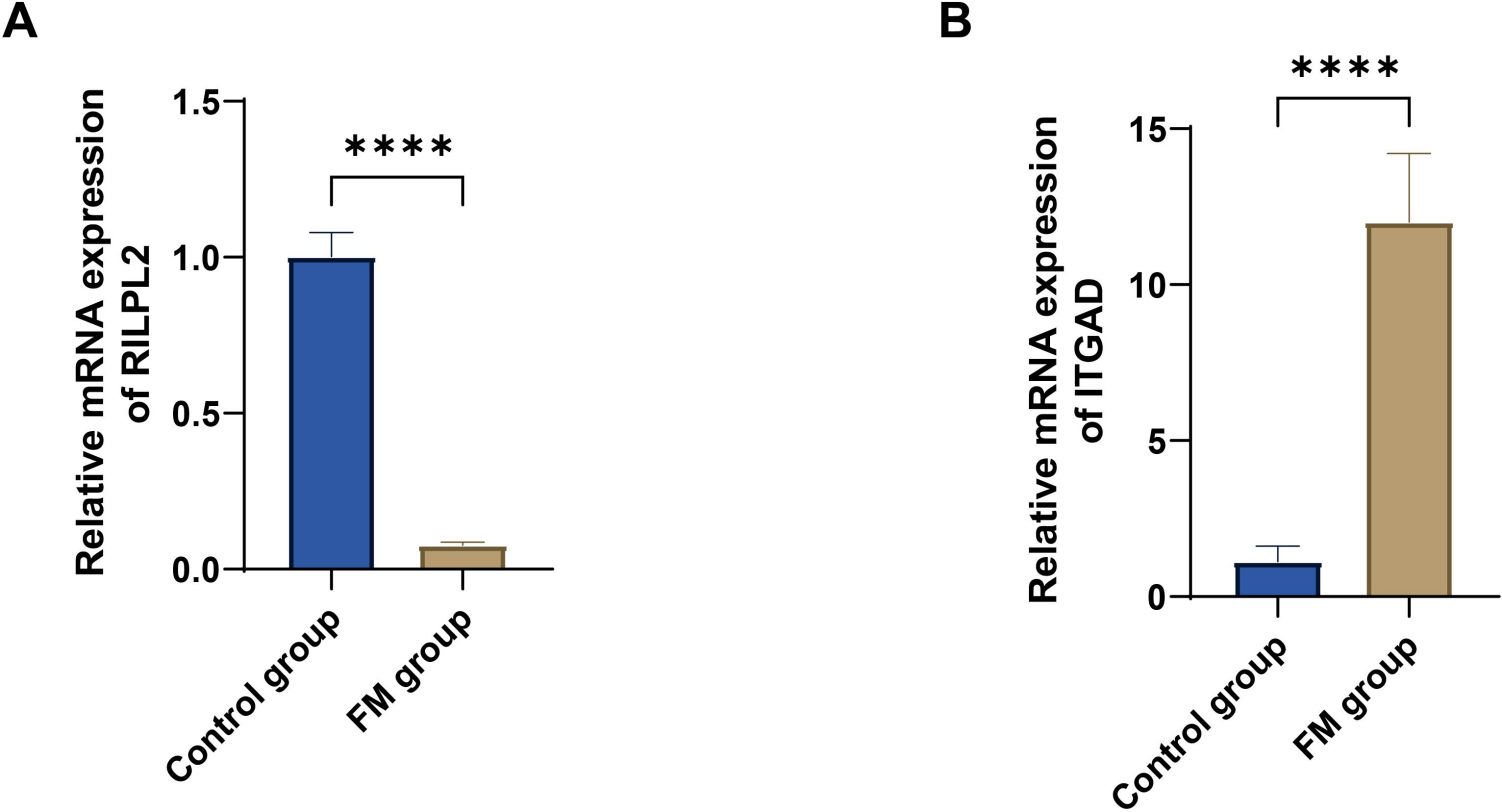
Based on qRT-PCR to verify the expression levels of RILPL2 and ITGAD in the FM mouse model. (**A**) Differential analysis of mRNA expression levels of RILPL2 in control and FM groups; (**B**) Differential analysis of mRNA expression levels of ITGAD in control and FM groups. The number of experimental mice used in the two groups was 6 mice per group. **** means *p* < 0.0001.

To further validate the expression of the 2 target genes (*RILPL2* and *ITGAD*) in FM, we constructed an FM model and determined the mRNA expression levels of these two genes using qRT-PCR. We observed a significant down-regulation of the mRNA expression level of *RILPL2* and a significant up-regulation of the expression level of *ITGAD* in gastrocnemius muscle tissues of FM mice relative to control mice (Figs. **8A** and **B**). These results suggest that the key markers we screened may have a potential impact on the occurrence and development of FM.

## 4 DISCUSSION

FM is a chronic pain syndrome with heterogeneity; its pathogenic mechanism remains disputable, and the current therapeutic efficacy is quite unsatisfactory [33]. Recent studies have shown that the functional alteration of the central/peripheral nervous system and immune system may be associated with FM progression [34, 35]. Andrés-Rodríguez *et al*. revealed that Mindfulness-Based Stress Reduction (MBSR) had notable immunomodulatory effects on FM patients, and some cytokines and chemokines might be the potential biomarkers for monitoring MBSR response [36]. Based on inflammation-related DEGs, Yao *et al*. classified FM patients into two subtypes. The innate immune response pathway was markedly activated in the micro-inflammation subtype, and 5 hub genes were screened to construct an accurate and reliable classification model [37]. In our present study, two molecular subtypes of FM were identified according to IRGs, the C1 subtype had a higher IRGs score than the C2 subtype, and 184 key module genes were obtained by WGCNA. Then, 11 hub genes (*TSPAN16*, *RILPL2*, *RASSF5*, *PGAP2*, *PADI2*, *NACC1*, *LRRC25*, *ITGAD*, *HIPK1*, *ATP6V0D1*, and *AP1M2*) were screened by LASSO regression analysis, which was utilized to construct a reliable diagnostic model. Moreover, 45 TFs targeting hub genes were predicted, and hub genes exhibited a strong correlation with immune cell infiltration and hallmark pathways.

*TSPAN16* is a member of tetraspanins involved in various activities, such as cancer, contagion, fertility, and the immune system [38]. *TSPAN16* could be applied as a biomarker for identifying male infertility [39], and was reported to be related to the overall survival of papillary thyroid carcinoma patients [40]. *RASSF5* belongs to a member Ras effector superfamily protein, usually playing an important role as a cancer suppressor [41]. In many malignant cell lines and primary tumors, the hypermethylation of promoter could inactivate *RASSF5* [42]. *PGAP2* participates in the lipid remodeling process of glycosylphosphatidylinositol-anchor maturation [43]. The gene mutation of *PGAP2* was known as the reason of “hyperphosphatasia, mental retardation syndrome-3” [44]. *PADI2* is a member of the peptidylarginine deiminase family. *PADI2* could facilitate the production of IL-1β, IL-6, and tumor necrosis factor (TNF)-α in macrophage, and targeting *PADI2* may become a new method for regulating inflammation response [45]. In rheumatoid arthritis patients, the single nucleotide variant of *PADI2* was found to be relevant to the susceptibility of interstitial lung disease [46]. *NACC1* encodes the nucleus accumbens-associated protein 1, primarily as a transcriptional co-regulator, exerts its biological functions regulating embryonic development, innate immunity, and related diseases [47]. *NACC1* is also involved in the development of chondrocytes and cartilaginous tissues [48]. *LRRC25* is a member of a leucine-rich repeat-containing protein family, which can negatively regulate the nuclear factor κB signaling pathway [49]. The deficiency of *LRRC25* could promote fibrosis and inflammation in cardiac hypertrophy [50]. *HIPK1* is a homeodomain-containing transcription factor modulating various cellular biological processes related to inflammation and stress response [51]. *ATP6V0D1* encodes the vacuolar ATPase and maintains the intracellular iron homeostasis [52]. In osteosarcoma (OS), *ATP6V0D1* was highly expressed in myeloid cells and osteoclasts, emphasizing its crucial role in OS patients [53]. *AP1M2* is an adaptor-associated protein complex and helps the construction of epithelium barrier functions [54]. In human cancers, the expression level of *AP1M2* was notably associated with patient outcomes and immune cell infiltration [55]. Noteworthily, in the GSE67311 and GSE221921 datasets, *ITGAD* was all highly expressed while *RILPL2* was all lowly expressed in FM samples. *ITGAD*, belonging to the membrane glycoproteins beta-2 integrin family, was discovered to be expressed in the tissues and circulating myeloid leukocytes [56]. In cell surface, high expression of *ITGAD* has a stable adhesivity with extracellular matrix proteins, resulting in the Macrophage reservation in inflammatory tissues [57]. This implies that the high expression of *ITGAD* in FM may be associated with macrophage adhesion and activation, which promotes the secretion of inflammatory factors and enhances the high immunoreactivity of the C1 subtype. In addition, Chen *et al*. found that *RILPL2* expression was significantly and positively correlated with CD4^+^ and CD8^+^ T cell infiltration in non-small cell lung cancer [58]. This also reveals that in FM, *RILPL2* may play a key role in maintaining immune homeostasis by regulating T cell function, and its low expression may also be associated with immune dysregulation in C2 subtype. Therefore, these hub genes may serve as potential diagnostic biomarkers for FM. There is still a need to validate the specific mechanism of action of these genes in immune regulation through more *in vivo* and *in vitro* experiments in the future, including further clarification of their functions and their associations with inflammatory pathways through knockout or overexpression experiments.

Furthermore, the correlation between 11 hub genes and the infiltration of 28 types of immune cells as well as hallmark pathways was analyzed. It was found that most hub genes were negatively associated with CD4/CD8 T cells while positively correlated with macrophage, mast cell, monocyte, and neutrophil, together with P53 pathway, IL6 JAK stat3 signaling, EMT, angiogenesis, and TGF beta signaling pathway. Research has shown that there are multiple layers of immune system dysfunction in FM, and T cells are related to the development and recession of chronic pain [59]. The serum level of CD8 T cells in chronic pain patients was lower compared with healthy controls [60]. The post-traumatic stress score of FM patients was negatively related to CD8 T cell numbers [61]. Macrophage and mast cells could activate microglia by secreting pro-inflammatory cytokines (IL-1β, IL-6, TNF-α) in FM [62]. The alteration of monocyte phenotype mediated by IL-5 was relevant to the pain outcomes in FM, and targeting IL-5 might be a promising strategy for intervening in FM [63]. The ratio of neutrophils/lymphocytes in the blood is known as a hallmark of inflammatory response [64]. The number of white blood cells, neutrophils, and lymphocytes in FM patients was markedly higher than in controls [65]. Besides, neutrophils could also secrete pain mediators directly or indirectly, and altering the interaction between Neutrophil activity and sensory neurons might be applied for targeting FM pain [66]. These findings manifested that the immune system may participate in the development and progression of FM, which needs further research.

## 5 LIMITATIONS

Nevertheless, several limitations of our study should be noticed. First, this study relied on transcriptome data from public databases. Despite the high quality of the data, the sample size was relatively small, which may limit the statistical validity and generalizability of the findings. Future studies should expand the sample size and utilize larger patient cohorts for validation. The representativeness and reliability of the results can be improved by collaborating with clinical centers to collect transcriptomic data from real patients and supplementing them with detailed clinical information. In addition, we will further validate the functions of key genes experimentally. For example, gene knockdown or overexpression experiments using CRISPR/Cas9 or RNA interference technology, combined with cellular function tests (*e.g.*, inflammatory factor secretion, immune cell function) and animal model experiments, can be used to explore the specific roles of these genes in FM. Finally, this study focused mainly on the expression analysis of individual genes. It did not explore the potential interactions among the 11 key genes and their roles in regulating the immune pathway. For this reason, future studies could investigate the interrelationships among these genes through co-expression network analysis and experimental validation (*e.g.*, co-immunoprecipitation experiments). In addition, the integration of proteomics and metabolomics data could further resolve gene interactions and their impact on FM pathology.

## CONCLUSION

In conclusion, our current study, based on some computational analyses, firstly identified 2 molecular subtypes of FM based on the IRGs, with varied enriched KEGG pathways. Then, based on the identified 2 molecular subtypes, WGCNA was applied to identify the 11 hub genes (TSPAN16, RILPL2, RASSF5, PGAP2, PADI2, NACC1, LRRC25, ITGAD, HIPK1, ATP6V0D1, AP1M2) with good diagnostic value, which were applied for the plotting of the TF-hub gene network using 45 TFs targeting these hub genes. Further immune infiltration analysis has revealed varied correlation between the hub genes and the immune cells infiltration and pathways. Besides, the molecular docking results highlighted the binding between chloroquine and NACC1 protein and L-citrulline and PADI2 protein, and the animal model has additionally validated the differed expression level of RILPL2 and ITGAD in FM animal model. These outcomes could provide a preliminary basis for understanding the pathogenesis and clinical diagnosis of FM.

## Figures and Tables

**Table 1:** Molecular docking binding energy.

Compound CID	Molecula_name	Gene_name	PDB_ID	Energy (kcal/mol)
2719	chloroquine	NACC1	3GA1	-5.4
9750	L-citrulline	PADI2	4n2c	-5.7

## Data Availability

The datasets generated and/or analyzed during the current study are available in the (GSE67311) repository, (https://www.ncbi.nlm.nih.gov/geo/query/acc.cgi?acc= GSE67311) and (GSE221921) repository, (https://www.ncbi.nlm.nih.gov/geo/query/acc.cgi?acc= GSE221921).

## LIST OF ABBREVIATIONS

ANOVA: Analysis of Variance
AUC: The Area Under the ROC Curve
BP: Biological Process
DEGs: Differentially Expressed Genes
EMT: Epithelial-Mesenchymal Transition
FM: fibromyalgia
GEO: Gene Expression Omnibus
GO: Gene Ontology
GS: Gene Significance
GSEA: Gene set Enrichment Analysis
IL: Interleukins
IRGs: Immune-Related Genes
KEGG: Kyoto Encyclopedia of Genes and Genomes
LASSO: Least Absolute Shrinkage and Selection Operator
MBSR: Mindfulness-Based Stress Reduction
MM: Module Membership
NES: Normalized Enrichment Scores
OS: Osteosarcoma
PCA: Principal Component Analysis
ROC: Receiver Operating Characteristic
ssGSEA: Single sample Gene Set Enrichment Analysis
SSS: Symptom Severity Scale
TF: Transcription Factor
TGF: Transforming Growth Factor
TNF: Tumor Necrosis Factor
WGCNA: Weighted Gene Co-expression Network Analysis
WPI: Widespread Pain Index
